# Towards a Greener Future: Sustainable Innovations in the Extraction of Lavender (*Lavandula* spp.) Essential Oil

**DOI:** 10.3390/foods14010100

**Published:** 2025-01-02

**Authors:** Sara Hedayati, Mohammad Tarahi, Arghavan Madani, Seyed Mohammad Mazloomi, Mohammad Hashem Hashempur

**Affiliations:** 1 Nutrition Research Center, School of Nutrition and Food Sciences, Shiraz University of Medical Sciences, Shiraz 7193635899, Iran; s_hedayati@sums.ac.ir; 2 Department of Food Science and Technology, School of Agriculture, Shiraz University, Shiraz 7144165186, Iran; tarahimohammad@yahoo.com; 3 Department of Food Hygiene Quality Control, School of Nutrition and Food Sciences, Shiraz University of Medical Sciences, Shiraz 7193635899, Iran; arghavan.madani@yahoo.com (A.M.); mazloomi@sums.ac.ir (S.M.M.); 4 Research Center for Traditional Medicine and History of Medicine, Department of Persian Medicine, School of Medicine, Shiraz University of Medical Sciences, Shiraz 7134845794, Iran

**Keywords:** lavender essential oil, medicinal herbs, extraction, distillation, emerging technologies, bioactivity, traditional Persian medicine

## Abstract

Lavender is one of the most appreciated aromatic plants, with high economic value in food, cosmetics, perfumery, and pharmaceutical industries. Lavender essential oil (LEO) is known to have demonstrative antimicrobial, antioxidant, therapeutic, flavor and fragrance properties. Conventional extraction methods, e.g., steam distillation (SD) and hydro-distillation (HD), have been traditionally employed to extract LEO. However, the low yield, high energy consumption, and long extraction time of conventional methods have prompted the introduction of novel extraction technologies. Some of these innovative approaches, such as ohmic-assisted, microwave-assisted, supercritical fluid, and subcritical water extraction approaches, are used as substitutes to conventional extraction methods. While other methods, e.g., sonication, pulsed electric field, and cold plasma, can be used as a pre-treatment that is preceded by conventional or emerging extraction technologies. These innovative approaches have a great significance in reducing the energy consumption, shortening the extraction time, and increasing the extraction yield and the quality of EOs. Therefore, they can be considered as sustainable extraction technologies. However, the scale-up of emerging technologies to an industrial level should also be investigated from the techno-economic points of view in future studies.

## 1. Introduction

Essential oils (EOs) are aromatic compounds obtained from different plant organs such as leaves, barks, peels, seeds, buds, flowers, and so on. These plant-derived compounds have been traditionally used around the world to promote health and treat various diseases [1,2,3]. Lavender (*Lavandula* spp.) belongs to the Lamiaceae family and is economically important for its EOs. Lavender, particularly lavender essential oil (LEO), has been found to offer flavoring, antioxidant, antimicrobial, anti-inflammatory, anticancer, antiemetic, anti-psoriatic, immunomodulatory, and wound-healing properties [4,5]. Additionally, its effectiveness in pain management, as well as in the treatment of a wide range of neuropsychiatric disorders such as depression, stress, and anxiety has been demonstrated by several researchers [6]. Because of these properties, LEO has a promising potential for use in food, pharmaceutical, textile, cosmetic, and other industries [7]. However, the fragrance and bioactivity of LEO might be affected by parameters such as species, maturity, origin, harvesting time, fertilization, extraction method and conditions [8]. Extraction methods such as steam distillation (SD) and hydro-distillation (HD) have been traditionally employed for the commercial scale production of EOs. Despite the simplicity of these methods, they are very time- and energy consuming. Also, the use of high temperature during extraction may damage the quality of EOs by decomposing the individual compound that mainly contributes to the bioactivity and fragrance properties [9,10,11]. On the other hand, the distillation systems consume fossil fuels or electrical energy that emits CO_2_ to the air. The amount of CO_2_ emitted into the atmosphere by human action is known as the carbon footprint, and several studies have demonstrated that conventional distillation methods have a higher carbon footprint in the environment [12]. Due to these limitations, the conventional extraction technologies of EOs are relatively unsustainable. Therefore, researchers and manufacturers are using cutting-edge alternative technologies that offer significant advantages over traditional extraction methods in terms of EO quality and quantity and environmental impact. Emerging technologies such as ultrasound-assisted, ohmic-assisted, microwave-assisted, cold plasma, pulsed electric field, supercritical fluid, and subcritical water extraction methods are gaining more popularity in the production of EOs [13]. These cutting-edge methods play a crucial role in enhancing the extraction yield and phytochemical quality of EOs, while decreasing the extraction period, solvent usage, energy consumption, and carbon footprint. Consequently, they contribute to the development of a more environmentally friendly and sustainable extraction process with reduced ecological impact [14,15]. This study comprehensively reviews the extraction mechanisms, advantages, and limitations of LEO extraction by conventional and emerging technologies from economic, physicochemical, and technological points of view.

## 2. An Overview of Lavender and Its Essential Oil

### 2.1. Origin and Cultivation

**Figure 1:**
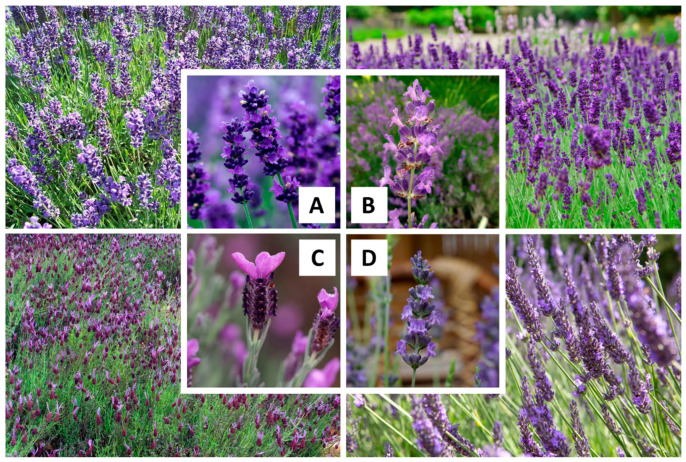
Photographs of four common lavender species: (**A**) *L. angustifolia*; (**B**) *L. latifolia*; (**C**) *L. stoechas*; and (**D**) *L. × intermedia*.

Lavender (*Lavandula* spp.) has been known worldwide for its medicinal properties since ancient times and has been used in various traditional medicines, including traditional Persian medicine. It is widely cultivated in the Mediterranean borderlands in poor, rocky soils, and in mild coastal climates from France, Greece, Italy, Spain, and Andorra to Morocco, Algeria, and Tunisia, although it is also grown in the Middle East (e.g., Iran and Saudi Arabia) and Southeast Asia (e.g., India and China). The genus Lavandula includes approximately 39 species, dozens of subspecies, and hundreds of hybrids, belonging to the Lamiaceae family [16,17]. Among the various identified species, *Lavandula angustifolia* Mill., *Lavandula stoechas*, *Lavandula latifolia* Medik., and *Lavandula × intermedia* Emeric ex Loisel. are the most common lavender species, mainly cultivated for their commercial EO production [18]. The images of these common lavender species are presented in Figure 1. *L. angustifolia* is a cold-resistant plant with colorful flowers which is generally referred to as English or fine lavender. On the other hand, *L. latifolia*, also known as spike lavender, is a grass-like lavender from the Mediterranean region. Additionally, *L. stoechasis* is a large plant with gray-green foliage and very strong-smelling flowers, commonly known as French or Spanish lavender. Moreover, *L. × intermedia* is a sterile hybrid between *L. angustifolia* and *L. latifolia*, which is also called lavandin [19,20]. In addition to the above-mentioned lavender species, *L. viridis*, *L. pedunculata*, *L. pinnata*, *L. multifida*, *L. pubescens*, *L. luisieri*, *L. lanata*, *L. gibsonii*, *L. heterophylla*, *L. dentata*, *L. coronopifolia*, *L. canariensis*, and *L. bipinnata* have also been widely used in the perfume, cosmetic, food, and pharmaceutical industries [21]. These species are annual herbaceous plants or small shrubs up to one meter in height with fragrant foliage and flowers that vary in growth habit, flowering period, morphology, and chemical composition. For example, *L. coronopifolia*, which is naturally found in the Arabian Peninsula, Western Asia, and North Africa, begins its flowering phase in January. In contrast, *L. pubescens*, which is native to Egypt, Israel, Jordan, Syria, Yemen, and Saudi Arabia, usually starts flowering between August and September. In addition, in recent years, there has been a trend of cultivating these species beyond their native habitats, which can cause differences in morphological characteristics, such as leaf and flower color, plant density, and seed weight, even in the same species. These differences in morphology, and consequently in the quantitative composition of individual compounds, can also be attributed to various climatic factors, such as exposure to sunlight, humidity, temperature, and altitude, as well as environmental variables, such as fertilization, soil type, and pH level [17,22]. According to data reported in 2017, Bulgaria stands as the leading producer of lavender globally, with about 4500 hectares of cultivated area that produce 200 tons of LEO per year. After that, France, Spain, and China are the world’s largest producers of LEO with 100, 80, and 40 tons per year, respectively [23,24]. In addition to the production of EO, these plants also have a landscape value, contributing to the tourist attraction of the region. Therefore, lavender cultivation can play an essential role not only in the global EO market but also in shaping the aesthetic appeal of different regions, which emphasizes the need for more attention in the future.

### 2.2. Phytochemical Compounds

Lavender contains a diverse array of chemicals, some of which are consistently present, while others are released in response to environmental stresses, such as changes in climate, disease, injury, and threats from predators [16,20]. EO is an economically appreciated compound in lavender, ranging from 1 to 3% of the plant’s dry weight, which is found in the oil glands between the sebaceous glands and the tiny hairs on the surface of the calyx [25]. The chemical profile of LEO can be analyzed using gas chromatography (GC), gas chromatography –mass spectrometry (GC-MS), high-performance liquid chromatography (HPLC), and nuclear magnetic resonance (NMR) spectroscopy methods. The characteristics of commercially used LEOs are well known, and the International Organization for Standardization (ISO) has published several quality standards for some lavender species, e.g., ISO 3054:2001 (lavandin Abrial EO, French type), ISO 3515:2002 (*L. angustifolia* EO), and ISO 8902:2009 (lavandin Grosso EO, French type) [20]. Generally, the chemical composition of LEOs is characterized by high amounts of oxygenated monoterpenes (e.g., linalool, limonene, borneol, linalyl acetate, camphene, camphor, and 1,8-cineole) and irregular monoterpenoids (e.g., terpinen-4-ol, fenchone, fenchol, lavandolol, and lavendolyl acetate), but low levels of monoterpene hydrocarbons (e.g., (Z)- and (E)-β-ocimene, p-cymene, α-pinene, and β-pinene), sesquiterpene hydrocarbons (e.g., β-farnesene and β-caryophyllene), and oxygenated sesquiterpenes (e.g., caryophyllene oxide) [23,26]. However, the amounts of these compounds may vary depending on the genotype, agronomic conditions, and harvest time, as well as the drying, extraction, and measurement methods. For instance, Cardia et al. [27] reported the EO of *L. angustifolia* cultivated in Brazil using the GC-MS and NMR techniques, with the predominance of 1,8-cineole (39.83%), borneol (22.63%), and camphor (22.12%). On the other hand, linalyl acetate is the most abundant component for the EO extracted from *L. angustifolia* grown in Bulgaria (27.5%), followed by linalool (24.1%), β-ocimene (7.0%), and terpinen-4-ol (5.1%) [28]. In another study, Uzkuç et al. [29] extracted LEO from Lavandula x intermedia var. Super A by microwave-assisted hydro-distillation (MAHD) and reported that linalool (29.0%), 1,8-cineole (13.9%), camphor (12.3%), and linalyl acetate (11.9%) were the major components of LEO, while linalool, 1,8-cineole, camphene, (Z)-ocimene, and 2,6-nonadienal were the main aroma-active compounds as determined by the gas chromatography–olfactometry (GC-O) method. In addition, Bogdan et al. [30] evaluated the chemical profile of EOs obtained from three different varieties of *L. angustifolia*, namely Moldoveanca 4, Vis Magic 10, and Alba 7. The results revealed that linalool, linalyl acetate, and terpinen-4-ol were the major identified constituents of these varieties, ranging from 32.19 to 46.83%, 17.70 to 37.13%, and 3.06 to 7.70%, respectively. Lavender species are also known for their high amounts of flavonoids (e.g., anthocyanins, flavones, isoflavones, flavanols, flavonols, flavanones, and chalcones), phenolic acids (e.g., cinnamic acid, rosmarinic acid, sagerinic acid, caffeic acid, chlorogenic acid, coumaric acid, hydro-p-coumaric acid, ferulic acid, ferulic acid-4-O-glucoside, salvianolic acid, isosalvianolic acid, 4-hydroxybenzoic acid, and 3,4-dihydroxybenzaldehyde), coumarins (e.g., herniarine and umbelliferon), sterols (cholesterol, β-sitosterol, stigmasterol, and campesterol), and minerals (e.g., Ca, K, Cu, Mn, Fe, and Zn) [16,22,23]. The presence of these diverse chemical compounds emphasizes the importance of lavender in promoting health and well-being, as well as the need for the continuous exploration and utilization of this remarkable botanical resource in various food and non-food products.

### 2.3. Potential Health-Related Benefits

The traditional uses of lavender species are widespread across various cultures and regions, highlighting their versatile medicinal properties. In the Mediterranean region, *L. stoechas* has been widely used in folk medicine to relieve flatulence, lung infections, inflammatory diseases, and central nervous system disorders, such as migraine and epilepsy. In addition, lavender species have analgesic, sedative, and antiseptic properties, making them valuable in wound care and the treatment of urinary tract infections and eczema [17]. Notably, historical records trace lavender’s medicinal use back to ancient times, with Greek botanist Dioscorides praising its digestive and analgesic properties, while the Romans valued it as an antiseptic. However, the therapeutic potential of lavender was recognized by the pioneering work of Gattefossé, a French chemist, in the early 1900s, who discovered its soothing effects and initiated the practice of aromatherapy. Subsequent studies have also expanded the health-promoting effects of lavender, indicating its efficacy in reducing stress, anxiety, and depression [31,32]. It is clear that due to the diversity of chemical compounds present in LEO, it is not possible to attribute these health-promoting effects to a specific mechanism of action. Generally, free radicals arise in the human body from both internal enzymatic and non-enzymatic cellular reactions, as well as external sources, such as radiation, pollution, and cigarette smoke. Their excess production disrupts the body’s balance, causing damage to proteins, lipids, and DNA, contributing to various diseases, such as hypertension, diabetes, and cancer. In this respect, LEO can exhibit high antioxidant activity by either preventing free radical formation or scavenging reactive oxygen species, donating hydrogen atoms or electrons. It can also inhibit oxidation processes by affecting enzymes, such as superoxide dismutase and catalase, or by chelating metal ions [22,33]. The collective evidence highlights the promising antioxidant potential of LEO, which may be useful in combating disorders related to oxidative stress and promoting overall health and well-being [34]. LEO is also known for its anti-inflammatory [35], analgesic [36], antidiabetic [37], antihypertensive [38], anticancer [39], antibacterial [40], antifungal [41], anti-ochratoxigenic [42], antiviral [43], anti-parasitic [44], and insecticidal [45] activities, as well as its antispasmodic and muscle relaxation effects [46], highlighting its potential as a natural therapeutic agent for different health conditions. Furthermore, the neurological effects of LEO have attracted much attention in recent years due to its interaction with various neuropharmacological targets, as evidenced by numerous in vitro and in vivo studies. LEO, primarily composed of linalool and linalyl acetate, exhibits anxiolytic, anti-depressive, and relaxing properties by modulating the GABA-A, MAO-A, and NMDA ionotropic receptors. Further research also suggests that LEO can exhibit neuroprotective effects by mitigating Aβ1-42 protein-induced neurotoxicity, which plays a key role in the formation of Alzheimer’s disease [47,48]. The ability of LEO to inhibit inflammatory responses, reduce histamine release, and enhance inhibitory tone in the nervous system contributes to its therapeutic effects on neurological disorders. On the other hand, aromatherapy with LEO has also shown promising results in reducing anxiety and enhancing mood levels in various clinical settings, including intensive care units (ICUs) and neurology wards. In this regard, studies suggest that the aroma of lavender can stimulate the autonomic nervous system, leading to relaxation and improved psychological well-being. Additionally, LEO aromatherapy has been associated with pain reduction and improved sleep quality in patients with conditions such as rheumatoid arthritis and dementia [16,49]. Overall, previous studies on the neurotherapeutic and aromatherapy effects of LEO emphasize its potential as a natural treatment for various nervous and anxiety conditions and highlight the importance of further exploration in these fields.

## 3. Extraction of Lavender Essential Oil

Choosing an appropriate extraction method and optimizing the extraction conditions can significantly improve the production yield and the quality of extracted LEO. Also, it can prevent the loss of pharmacological and flavor compounds and improve the physicochemical and biological properties of the extracted EOs. LEO can be extracted by several techniques categorized into two main groups, namely conventional/classical and innovative/advanced extraction methods, which are comprehensively reviewed in this section.

### 3.1. Conventional Extraction Methods

The conventional EO extraction approaches include SD, HD, hydrodiffusion, solvent extraction (SE), and cold pressing. Hydrodiffusion is only used for dried plants that are heat sensitive and cannot withstand boiling temperatures. The extraction is accomplished at low pressure or under vacuum, and the steam temperature is set under 100 °C. In the hydrodiffusion process, dried plants are placed in a container and steam is applied to the container from the top of a generator [50]. SE is another classical method that is commonly used in the perfume industry to extract EOs from delicate plant tissues, such as flowers, which cannot be exposed to heat or steam. The plant materials are macerated in organic solvents, e.g., ethanol, methanol, hexane, acetone, or petroleum ether, and mildly heated; afterwards, the mixture passes through a filter and the solvent is evaporated [51]. The obtained filtrate is a blend of EO, resin, wax, and fragrance. To isolate the EOs from the filtrate, the mixture is blended with alcohol and distilled at low temperatures. Alcohol absorbs the fragrance and then evaporates, but the EOs remain in the flask [52]. The EOs obtained by SE may contain toxic solvent residues; thus, they cannot be used for food applications. However, if ethanol is used as the solvent, it is regarded as safe, and the extracted EO can be used as a food-grade ingredient [53]. SE is not extensively used for EO extraction due to its complexity, expensiveness, high solvent consumption, time-consuming nature, and unsatisfactory reproducibility. Cold pressing is traditionally used to extract citrus-peel EOs. In this process, EOs are removed mechanically from the oil glands/oil sacs in the external part of the mesocarp by cold pressing. As a result, the oil sacs are broken and an emulsion is released from the citrus zest. Subsequently, the emulsified EOs are recovered by centrifugation [54]. HD is one of the simplest and oldest techniques for the extraction of EOs. This method was invented by Avicenna, who extracted pure rose EO for the first time [55]. HD can be performed by the Clevenger method or simple SD. In the Clevenger method, the plant material and water are heated and the EOs are evaporated in the apparatus. Subsequently, the steam and EOs are cooled until they condense, and the EOs are isolated from the water in a separating funnel. In contrast, in simple SD, steam passes through plant materials and carries away the EOs. The steam and EOs are then cooled and turned back into liquid. The EOs are then isolated from the water using a separating funnel. In both the Clevenger and simple SD methods, two layers are produced in the separating funnel: an oil (i.e., EOs) layer on the top, and a water layer at the bottom [56].

Several studies investigated the influence of different parameters such as lavender variety, drying method, and, extraction conditions on the quantity and quality of LEO extracted by conventional methods. In this regard, Bogdan et al. [30] compared the chemical composition as well as the antimicrobial and antioxidant activities of EOs from the inflorescences of three varieties of *Lavandula angustifolia* Mill. (i.e., 3-Alba 7, 2-Vis Magic 10, and 1-Moldoveanca4). The lavender flowers were harvested in three consecutive years in June from the northern part of Romania, and LEO was extracted by HD for 1h at 100 °C. The composition of LEO was analyzed by GC-MS and FT-IR spectroscopy. The major compounds were as follows: linalool, linalyl acetate, and terpinen-4-ol. The antioxidant capacity of the LEOs were evaluated by the 1,1-diphenyl-2-picrylhydrazine (DPPH) and 2,2′-azino-bis(3-ethylbenzothiazoline-6-sulfonic acid) (ABTS) assays, and the highest activity was determined for var. Alba 7. The antimicrobial activity of EOs was evaluated by the disk diffusion method and minimum inhibitory concentration (MIC) against Staphylococcus aureus, *Pseudomonas aeruginosa*, *Candida albicans*, and *Escherichia coli*. The results revealed that LEOs obtained from all of the varieties have substantial bactericidal activity against *E. coli* and *S. aureus*, and antifungal effects against *C. albicans*. However, the best antimicrobial results were obtained for var. Vis Magic. Also, it was found that none of the extracted EOs were effective against *P. aeruginosa*. In another study, Zagorcheva et al. [57] obtained LEO and volatile compounds from the inflorescence of seven Bulgarian lavender cultivars by organic solvent (hexane) extraction. The composition of LEO was determined by GC/MS analysis, and a total of 32 volatile compounds were identified. It was observed that the composition of volatiles and EOs were significantly influenced by the lavender cultivar. Furthermore, Jianu et al. [58] determined the composition and antimicrobial activity of EOs from the inflorescences of lavender and lavandin extracted by SD. The plant materials were harvested in 2011 in Romania and analyzed by GC-MS. The major compounds in the EOs of lavender were caryophyllene (24.1%), beta-phellandrene (16%) and eucalyptol (15.6%), whereas lavandin EO contained camphor (32.7%) and eucalyptol (26.9%) as the main components. The EOs exhibited antimicrobial properties against *S. aureus*, *S. flexneri*, *E. coli*, and *S.* Typhimurium; however, they were not effective against *S. pyogenes*.

The extraction time significantly affects the composition, physicochemical properties, and bioactivity of LEO. Several studies have investigated the effect of extraction time on the quantity and quality of LEO. In this regard, Wesolowska et al. [59] studied the effect of SD time on the content and composition of the EO isolated from lavender. Initially, 5g of lavender flowers were added to a flask filled with 500 mL of distilled water and subjected to SD for 40 min, 1 h, or 2 h. The highest LEO yield (2%) was extracted after 2 h of SD, while the lowest LEO percentage (1%) was extracted after 40 min of distillation. The GC-MS analysis results revealed that the isolated LEO contained 28.78–30.68% linalool, 12.35–17.67% linalyl acetate, and 7.57–11.49% α-terpineol as the major components. Also, it was demonstrated that the highest linalool content (30.68%) and linalyl acetate content (17.67%) were isolated after 1 h of SD. Meanwhile, the highest α-terpineol content (11.49%) was extracted after 40 min of SD. Similarly, Zheljazkov et al. [60] performed a study to evaluate the influence of distillation time on LEO composition and yield. The EOs were extracted by SD for 1.5, 3, 3.75, 7.5, 15, 30, 60, 90, 120, 150, 180, or 240 min. The extraction yield ranged from 0.5 to 6.8% and the maximum LEO yield was obtained after 60 min. The concentration of individual compounds was significantly affected by the distillation time. The cineole content was between 6.4 and 35%, and fenchol ranged from 1.7 to 2.9%. The highest concentration of these components was extracted after 1.5 min, but decreased with increasing the extraction time. The camphor content ranged from 6.6 to 9.2% and the maximum concentration was extracted at 7.5–15 min of distillation, whereas the linalool acetate content ranged from 15 to 38% and the maximum content was obtained at 30 min of extraction. The obtained results suggested that extraction yield did not increase after 60 min. The changes in LEO yield and the concentrations of individual compounds during extraction time provided a good fit to the asymptotic nonlinear regression model. In another study, Hristova and Veleva [61] investigated the effect of extraction time on the extraction yield of LEO from the *Seuthopolis* variety. The plant material (100 g) was immersed in distilled water (500 mL) and LEO was extracted by HD using a Clevenger apparatus for 35, 40, 45, 50, 55, 60, or 65 min. The maximum LEO yield was extracted after 60–65 min and no significant differences were detected between the extraction yields at 60 and 65 min. Therefore, the optimum LEO extraction time was 60 min. In a further study, Tarakemeh et al. [62] studied the effect of extraction time (1 and 3 h) and drying method (shade drying, sun drying, and oven drying at 45 °C) on the yield and the chemical composition of LEO. The dried lavenders were exposed to HD with a Clevenger apparatus to extract LEO according to the European Pharmacopoeia method (third edition). The results of the GC and GC/MS analyses revealed that the composition of LEO was not affected by extraction time, whereas the drying methods significantly influenced the LEO composition, and shade drying resulted in the highest LEO quantity and linalool concentration.

In addition, Zheljazkov et al. [63] conducted a study to evaluate the composition of LEO extracted by SD from fresh and dried plants. Aliquots of 500 g fresh or 250 g of dried lavender flowers were added to 2 L of water and the EOs were extracted for 60 min. LEO content varied from 0.71 to 1.3% and the extraction yield increased with the extraction time. The major compounds of LEO were linalool and linalyl acetate. The EOs extracted from dried lavender had higher amounts of linalyl acetate and caryophyllene but lower contents of myrcene compared to the EOs from fresh lavender. Moreover, Wainer et al. [64] compared the effectiveness and profitability of LEO extraction by different conventional approaches to determine the best method for the commercial-scale extraction of LEO. The EOs were extracted by SD, HD, and cellulase-assisted HD. The extraction time for SD, HD, and cellulase-assisted HD were 57, 51, and 49 min, and their average energy consumption was 15.0, 13.4, and 30.8 kJ/g, respectively. The quality of LEO produced by cellulase-assisted HD was better than other samples, as it had a more pleasant smell. The LEO extracted by cellulase-assisted HD presented the lowest camphor content, while the SD oils showed the highest camphor content. On the other hand, the cost analysis results revealed that HD is the most profitable, and cellulose-assisted HD is the least profitable method for LEO extraction. Also, HD showed a reasonable extraction period and the lowest energy consumption. It is a simple method with a basic setup that can easily be scaled up. The LEO obtained by HD had a high quality, and a pleasant smell. Based on the obtained results, HD was recognized as the best technique for the commercial production of LEO. However, the quality and odor of LEO extracted by cellulase-assisted HD was outstanding, and if a low-cost enzyme could be used, this method could be considered as an economic method. Therefore, further research on cellulase-assisted HD would be worthwhile. In another study, Keivanfar et al. [65] investigated the effect of HD and SD extraction methods on the antioxidant properties of lavender, rosemary, and *Ferulago* EOs. For the HD method, 300 g of powdered plant materials were mixed with 1200 mL of distilled water in a boiling flask and the EOs were extracted using a Clevenger apparatus for 3 h. Meanwhile, in SD, 310 g of lavender, rosemary, and *Ferulago* and 1240 mL of distilled water were added to the boiling flask and the extraction was accomplished in 3 h. The total phenol content (TPC), total flavonoid content (TFC), bioactive compound yield, and antioxidant activity of EOs were determined. The results indicated that SD resulted in a significantly higher (*p* < 0.05) extraction yield of bioactive compounds than HD. Also, the phenol and flavonoid contents and the antioxidant activity of EOs isolated by SD were higher than HD. Mathematical models can be used to determine the effect of different extraction parameters on the efficiency and the chemical composition of EOs and optimize the extraction conditions. In addition, Cassel et al. [66] used a mathematical model based on single-plate particle description to predict the recovery of EOs from lavender by SD and compared the results with the experimental data. The apparatus comprised a boiler that was heated by an electric resistance, an extraction chamber, and a Clevenger trap. The LEO extraction curve fitted well with the experimental data, and the maximum yield was 0.32%. In another study, Perović et al. [67] modeled the kinetics of the LEO extraction yield by HD with a phenomenological model. The main objective of this study was to assess the effects of HD time and the hydro-module on LEO extraction yield to optimize the process conditions for the maximum extraction of LEO. A full factorial design in combination with response surface methodology (RSM) were used for optimization. Initially, 15 g of lavender flowers were submerged with water (10–25 mL·g^−1^) in the Clevenger vessel and heated with an electrical heater. HD was performed for 5–240 min and the extracted LEO was collected in the measuring tube to determine its volume. In the early stages of HD, fast LEO distillation was observed, while, after the occurrence of saturation, slow diffusion stage was detected, indicating the suitability of using phenomenological model. The statistical modeling results proved that the hydro-module had a more significant effect on the yield of LEO compared to HD time. Also, it was found that the hydro-module had a negative effect on the extraction yield, while the distillation time had positive effects on the LEO yield. The optimum conditions for LEO extraction by HD were an extraction time of 108 min and a hydro-module of 10 mL·g^−1^, which resulted in an extraction yield of 5.60 mL·100 g^−1^. The application of these models is very effective in increasing the extraction efficiency and reducing the total cost of LEO.

### 3.2. Emerging Extraction Methods

Conventional extraction methods have disadvantages such as expensive operational costs, long extraction time, and high processing temperature. Also, the problems with conventional extraction techniques lie in the low efficiency and large amounts of plant materials needed to extract EOs on a commercial scale. For instance, to conventionally extract 1 kg of LEO, 200 kg of fresh lavender flowers are needed, which leads to an increase in the total price of natural LEO [68]. Therefore, EO producers must use more effective and efficient extraction methods to improve profitability and create sustainable competitive advantages. Additionally, applying high temperatures for a long time in conventional extraction methods may cause oxidation, hydrolysis, or the isomerization of the EO components. These chemical changes greatly damage the quality of EOs. Therefore, the development of high-efficiency mild extraction methods that produce high-quality EOs is highly needed. In response to this need, several emerging extraction methods have been developed, which will be discussed in this section.

#### 3.2.1. Supercritical Fluid Extraction

**Figure 2:**
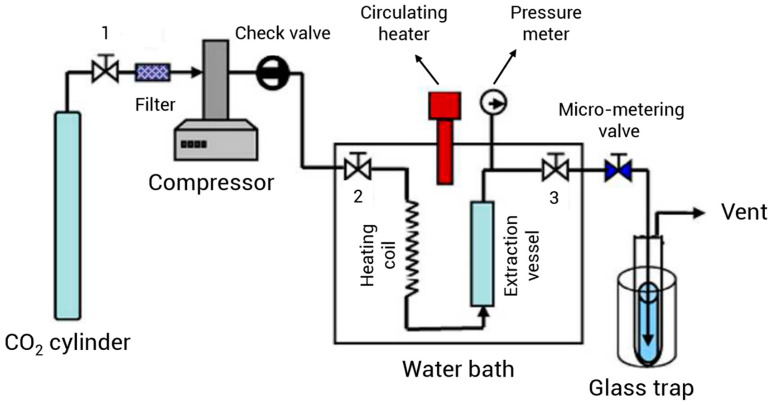
A schematic diagram of the supercritical CO_2_ extraction technique. The numbers 1, 2 and 3 indicate the stopping valves; adapted from Danh et al. [71].

Supercritical fluid extraction (SFE) is the process of using supercritical fluids as extracting solvents to isolate EOs from plant tissues. SFE is a suitable alternative to conventional extraction processes for the production of EOs, because it is a fast and cost-effective extraction method that has the potential to be used on an industrial scale (Table 1 and Figure 2). In a study, the SFE of LEO, considering its economic applicability, was evaluated by Cruz-Sánchez et al. [69]. SFE was performed at a temperature of 60 °C, pressures of 180 or 250 bar, and different volumes (20, 50, and 100 L), and a maximum yield of 6.9% was achieved. The selling price of LEO for the pilot-scale (20 L) and industrial-scale (50 and 100 L) extraction was considered to be 1.38 EUR/g and 0.9 EUR/g, respectively, and the profitability of LEO extraction by SFE was verified using financial analysis for eight years. Additionally, the price of equipment, which significantly impacted the economic feasibility, was appropriately evaluated in this study. An assessment of the financial ratios led to a return on equity (ROE) value exceeding 57% in all cases, highlighting that producing LEO by SFE technology is an attractive investment opportunity on an industrial scale. Additionally, SFE offers a high extraction efficiency, prevents the thermal and hydrolytic decomposition of EO components, and the EOs extracted by this method have a heavier smell compared to the EOs obtained by other extraction methods [70,71,72]. Supercritical carbon dioxide (SC-CO_2_) is one of the most widely used materials in this extraction approach. Recent advances in SC-CO_2_ extraction have shown that it can be a promising alternative to classical extraction systems [73,74] because it is non-toxic, inflammable, inert, and easily reaches its critical point [75]. Furthermore, its high solvent power can be easily controlled by changing the pressure and temperature. Therefore, SC-CO_2_ is considered as an appropriate and green option for the extraction of EOs. SC-CO_2_ is suitable for the extraction of lipophilic compounds; however, the main problem with SC-CO_2_ is its low polarity, which reduces the solubility of polar compounds. To improve the polarity of SC-CO_2_, a co-solvent such as isopropanol, ethanol, acetonitrile, or ethyl acetate can be used [76,77].

In a study by Cruz-Sánchez et al. [69], the SC-CO_2_ and Soxhlet extraction of LEO were compared. The SC-CO_2_ extraction process was accomplished at different temperatures (40–60 °C), and pressures (180, 250 and 300 bar), with or without co-solvents (ethanol and ethyl acetate). The effects of the extraction conditions on the antioxidant capacity and the chemical composition of LEO were determined. For the Soxhlet extraction, 20 g of lavender flowers were placed in a filter paper cartridge and 250 mL of hexane was poured into the flask and heated to reach the boiling point of hexane (70 °C); the vapor condensed and fell into the cartridge to extract the LEO. After 3 h of extraction, hexane was separated from the LEO by a rotary evaporator. In the supercritical extraction (SCE), SC-CO_2_ passed through lavender flowers and dissolved the extractable constituents. Then, the pressure was reduced and the LEO and the solvent were separated. The solubility of LEO in SC-CO_2_ was measured by high-pressure variable volume and modeled with semi-empirical models. The results showed that the highest extraction yield and solubility of LEO were obtained at 60 °C and 250 bar. Also, the application of 0.2% *v*/*v* ethanol, as a co-solvent, significantly improved the extraction yield of LEO. The major compounds of LEO in the samples extracted by Soxhlet and SC-CO_2_ were linalool and linalyl acetate. However, the amounts of these compounds were significantly different. The Soxhlet extraction yielded higher linalyl acetate (49.55 vs. 43.03) and lower linalool (27.76 vs. 32.20) compared to the SC-CO_2_ extraction.

To obtain the highest LEO extraction yield with appropriate amounts of useful components, the SC-CO_2_ extraction conditions should be optimized. In a study by Akgün et al. [78], EOs from lavender flowers (*Lavandula stoechas* L.) were extracted by a semi-continuous SC-CO_2_ extractor under varying operating conditions, including different pressures (8–14 MPa), temperatures (308–323 K), and CO_2_ flow rates (1.092–2.184 × 10^−3^ kg/min). The experimental results indicated that LEO extraction rate was not significantly influenced by the CO_2_ flow rate. However, a linear increase was observed in the extraction rate as a function of time in the early stages of extraction. The extraction speed was increased with the increase in temperature due to the increased solubility of LEO compounds as a result of high vapor pressure. In addition, the extraction process was modeled using a quasi-steady-state model with an adjustable parameter, namely the intraparticle diffusion coefficient. The modeling results showed that only one parameter (intraparticle diffusion coefficient (effective diffusivity) De) was adjustable in this model. In a similar study, the extraction of LEO was accomplished by SC-CO_2_, and the effects of operating conditions on the overall relative efficiency were calculated with respect to SD. The extraction parameters such as pressure (70–110 bar), temperature (30–50 °C), CO_2_ flow rate (7.2–36 L/h), and the plant material particle size (140–2500 μm) were optimized by RSM. The third-order model based on central composite design satisfactorily predicted the experimental results of the SC-CO_2_ extraction. The results revealed that the optimal conditions to obtain the highest extraction yield were a pressure of 85.77 bar, a temperature of 36.58 °C, a carbon dioxide flow rate of 10.11 L h^−1^, and a particle size of 2143 μm. Also, GC-MS analysis revealed that the main components of LEO included fenchone and camphor, which is different from the other studies. This significant dissimilarity in the major compounds of LEO can be attributed to the differences in plant material and extraction conditions [79].

The LEO obtained by SC-CO_2_ has higher amounts of bioactive and useful compounds. Therefore, it has the potential to be used in biomedical products. Avsar et al. [80] extracted LEO using a SC-CO_2_ extraction system under the pressure of 100 bar and a temperature of 40 °C. The chemical compounds of the extracted EOs were determined by GC-MS analysis. The antioxidant capacity of the extracted LEO was examined by DPPH radical assay, and the antimicrobial activity was evaluated against various microorganisms, including *K. pneumoniae*, *B. subtilis*, *E. coli*, *P. aeruginosa*, *S. aureus*, and *S. pneumoniae*. The results demonstrated the substantial antimicrobial and antioxidant properties of LEO. The extracted LEO was used in a topical cream, and it was found that the samples were consumable even after 6 months of storage. Therefore, the LEO extracted by SC-CO_2_ can be used as a bio-preservative in creams and cosmetic products to ensure long-term use without compromising consumer skin health. A study by Danh et al. [71] investigated the effects of temperature, pressure, and time on the SC-CO_2_ extraction of lavender essential oil. The results revealed that time and pressure had a significant linear impact on the antioxidant activity and extraction yield of LEO. However, temperature, except for its interaction with pressure, had a lesser effect on the extraction yield. The LEO obtained under high temperature and pressure, as well as an extended extraction time, exhibited higher antioxidant activity and yield. Overall, the temperature effects were partially quadratic, showing a second-order effect and interaction with pressure. These parameters did not have a significant effect on LEO composition, and linalool, linalyl acetate, borneol, and camphor were the major compounds, comprising approximately 80% of the identified components. Researchers determined that the optimal conditions for achieving the highest antioxidant capacity (75.8%) were 53.4 °C, 190.8 bar, and 73.6 min. Additionally, the estimated yield under optimal conditions was 13.2% at 53.4 °C, 207.2 bar, and 56.3 min. Interestingly, a comparable performance (11.1%) was also estimated under milder extraction conditions of 36.6 °C, 100 bar, and 73.6 min. Zanotti et al. [81] extracted LEO by SC-CO_2_ under a pressure of 8 megapascals, a temperature of 40 °C, and the CO_2_ mass flow rate of 0.60–1.50 kg/h. They found that at higher flow rates (0.90 to 1.50 kg/h), the LEO yield was 5.2% *w*/*w*, while it was significantly lower (2.3% *w*/*w*) at lower flow rates. The extracted LEO was wax-free, indicating a successful distillation and containing τ-cadinol, lavandulol, β-caryophyllene, viridiflorene, isocaryophyllene, cedrenalol, linalool, and 1,8-cineol. The amount of these compounds was 13, 10.5, 10, 8.5, 6, 4.5, 4, and 4%, respectively. Kamiie et al. [82] compared the effectiveness of SFE and HD for LEO extraction. The results revealed that the HD method had the capability to extract 66 compounds, whereas SFE extracted 46 compounds. The major components obtained through the HD method were linalyl acetate, terpinen-4-ol, and linalool, with respective percentages of 25.3%, 16.4%, and 13.0%. In contrast, the essential oil constituents obtained through the SFE method were as follows: linalyl acetate (30.6%), terpinen-4-ol (14.1%), and lavandulyl acetate (8.4%). However, the extraction efficiency of LEO using the SFE technique was at least 10 times higher than that achieved with the HD method. Additionally, the results revealed that the SFE is more appropriate than HD for obtaining compounds that are relatively unstable at higher temperatures. In a study conducted by Ghoreishi et al. [83], LEO was extracted using SC-CO_2_ with both a periodic static–dynamic (PSD) method and a conventional semi-continuous (SC) method. In the SC process, both dynamic and static extraction were carried out only one time, whereas the PSD method involved repeated static–dynamic extraction cycles. In the PSD approach, the static extraction was performed for a specific period of time, followed by the dynamic extraction in which the residence time of the extraction column was 2 min (at a fixed CO_2_ volumetric flow rate of 2 mL·min^−1^). The extraction efficiency in the PSD method was estimated to be 94.4%, while it was 90% in the SC technique. These findings indicated that SC-CO_2_ is suitable for separating components such as linalyl acetate, linalool, fenchone, and camphor. Furthermore, the use of the PSD process resulted in a substantial reduction in energy consumption and solvent usage compared to SC method. A study conducted by Babu et al. [84] compared the quality and quantity of LEO extracted by SC-CO_2_ and HD. The results showed that HD yielded a higher efficiency (2.5%) compared to SC-CO_2_ (0.6–1.8%). However, SC-CO_2_ extraction produced higher-quality EOs in terms of higher concentrations of linalyl acetate and β-caryophyllene. In SCE, the operating conditions had significant effects on the yield, composition, and physicochemical properties of LEO. Increasing the density of SC-CO_2_ led to improved performance. In the HD method, the EOs contained higher levels of alcohols, monoterpene hydrocarbons (MHs), and monoterpene cyclic ethers (MCEs), while the predominant components of SC-CO_2_ EOs were esters and sesquiterpenoids. The optimal extraction conditions for LEO quality and yield are SC-CO_2_ at 12 and 14 MPa and temperatures of 35 and 45 °C. Meanwhile, the lowest LEO extraction yield, with the highest concentration of esters and the lowest amounts of MCE, alcohol, and carbonyl, was obtained at 10 MPa and 55 °C. The results highlighted that the diversity in LEO quality and the variations in the aromatic compounds may be attributed to the differences in the physicochemical properties of solvents, the changes in the solvation power of carbon dioxide, and the other thermo-physical attributes of the solvents or solutes under process conditions. In a study by Danh et al. [74], the impact of three extraction methods, HD, SC-CO_2_ extraction, and organic solvent (hexane) extraction, on the composition, yield, antioxidant and antimicrobial activities of LEO were evaluated. In SC-CO_2_, the extraction yield was approximately 6.7%, which was comparable to the yield obtained by hexane extraction (7.6%) but meaningfully greater than that of water distillation (4.6%). Approximately 80% of the identified components in all LEO samples consisted of linalool, linalyl acetate, camphor, and borneol. The hexane extraction produced EOs containing high amounts of undesirable compounds such as waxes, pigments, and semi-solid albumin-like substances that were not observed in the LEO samples obtained by SC-CO_2_ and HD. On the other hand, the EOs extracted by HD showed thermal degradation and the loss of some aromatic compounds. The SC-CO_2_ EOs exhibited a similar aroma profile to the raw material and showed minimal heat-induced degradation. Additionally, their antioxidant activity was significantly higher than the EOs obtained by HD and hexane extraction. In terms of antimicrobial activity, the EOs produced by SC-CO_2_ and HD were more effective against *E. coli*, *E. faecalis*, *S. aureus*, and *C. albicans* compared to the hexane extracted LEO. Zhi-ling et al. [70] extracted LEO by microwave-assisted extraction (MAE) and SC-CO_2_ and analyzed the volatile oil contents of both LEO samples by gas chromatography. The GC analysis identified 84 components in the LEO sample obtained by MAE and 65 components in the EOs extracted by SC-CO_2_. The LEO obtained by SC-CO_2_ was free from impurities and residual solvents and had a heavier smell compared to the LEO produced by MAE. The extraction time, pressure, and temperature significantly influenced the efficiency of the SC-CO_2_ method, and the optimal conditions were as follows: a temperature of 45 °C, a pressure of 22 MPa, and an extraction time of 1 h. Under these conditions, the yield was 4.80%, while it was 3.67 for MAE. In the study conducted by Reverchon et al. [85], LEO was obtained by SCE and HD, and its composition was analyzed by the GC-MS technique. The optimal extraction conditions in the SC-CO_2_ were a pressure of 90 bar and a temperature of 48 °C. The most significant difference between the EOs extracted by SC-CO_2_ and HD methods was observed in the content of linalyl acetate, which was estimated to be 34.7% in the SC-CO_2_-extracted LEO and 12.1% in the HD-extracted LEO. These findings suggest that linalyl acetate is partially decomposed during HD. Additionally, the sensory evaluation results showed that the LEO obtained by HD exhibited a noticeable difference in aroma from the original lavender scent, whereas the fragrance of the SC-CO_2_-extracted LEO was similar to the initial plant material. A study was conducted by the authors of [73] to compare the composition and yield of LEO extracted by SCE and HD. In the HD method, the primary compounds of LEO included j-pinene (35.9%) and lavandulyl acetate (14.1%). Meanwhile, the SFE method yielded linalyl acetate (73.5%) and lavandulyl acetate (7.5%) as the main constituents, suggesting that the extraction method significantly affects the therapeutic properties of the produced EOs. According to the results, the efficiency of the SFE method was approximately six times higher than that of HD, and the quality of LEO extracted under optimized SFE was estimated to be much higher than the HD-extracted EOs. Kiran Babu et al. [86] assessed the qualitative and quantitative characteristics of lavender volatile fractions extracted by SE, SCE, water distillation (WD), and water–steam distillation (WSD). The highest yield was obtained by WD (1.2%), followed by WSD (1.12%), SE (0.8%), and SCE (0.5%). The key compound determining the quality of lavender aroma is linalyl acetate. SCE produced a superior-quality, ester-rich fragrance with minimal undesirable compounds compared to conventional production methods. The extraction percentages for linalool were as follows: SCE (51.8%), SE (31.4%), WSD (31.4%), and WD (26.8%). Additionally, linalool levels were highest in the following order: WD (30.9%), SCE (23%), WSD (20.5%), and SE (17.3%). Undesirable compounds in lavender aroma, such as coumarin and 7-methoxycoumarin, were present in significant amounts in SE (20.5% and 11.2%, respectively), but were not detected in other extraction methods. In cosmetic and flavor industries, LEO with higher amounts of ester is preferred. Consequently, the choice of extraction method can be determined based on the intended final application of the EOs. Generally, LEO from SC-CO_2_ extraction is free of impurities with high yield and quality. Therefore, SC-CO_2_ extraction is an appropriate process for the industrial production of LEO.

**Table 1:** The effects of species, cultivation area, and supercritical CO_2_ extraction conditions on the yield and composition of lavender essential oil.

Species	Cultivation Area	Extraction Conditions					LEO Yield (%)	Main Components	Reference
		P (bar)	CO_2_ Flow Rate	T (°C)	Time (min)	Particle Size (mm)			
*Luvandula stoechas*	Ömerler village, Turkey	1	NS	20	20	2.0–2.5	1.98	Fenchone and camphor	[79]
*Lavandula stoechas*	Aydin-Ҫine region, Turkey	100	1.46 mL/min	35	180	1.2	1.2	Camphor (43.74%) and fenchone (33.14%)	[78]
*Lavandula officinalis*	Mersin region, Turkey	145	NS	45	NS	NS	4.68	Linalool and linalyl acetate	[80]
*Lavandula hybrida*	Isfahan, Iran	108.7	5 mL/min	48.5	144	0.60–0.85	4.77	Linalool and linalyl acetate	[87]
*Lavandula angustifolia*	Himachal Pradesh, India	110	60 g/min	35	120	NS	1.8	Linalyl acetate and linalool	[84]
*Lavandula angustifolia*	Victoria, Australia	145	2 mL/min	45	40	0.8	6.7	Linalool and linalyl acetate	[74]
*Lavandula angustifolia*	Mostar, Bosnia and Herzegovina	100	2.3 kg/h	41	90	0.36	7.28	Linalyl acetate and linalool	[88]
*Lavandula angustifolia*	Hirosaki, Japan	120	27 L/h	40	120	NS	13.1	Linalyl acetate and terpinen-4-ol	[82]
*Lavandula angustifolia*	Himachal Pradesh, India	250	NS	50	45	NS	0.5	Linalyl acetate and linalool	[86]
*Lavandula angustifolia*	Tehran, Iran	200	10 mL/min	45	90	NS	2.5	Linalyl acetate and lavandulyl acetate	[73]
*Lavandula angustifolia*	Warszawa, Poland	250	NS	60	45	NS	9.2	NS	[89]

P: pressure; T: temperature; NS: not stated.

#### 3.2.2. Subcritical Water Extraction

**Figure 3:**
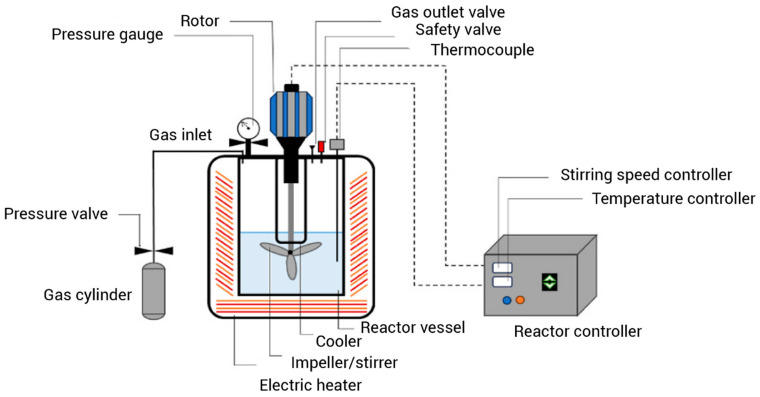
A schematic diagram of the subcritical water extraction system; adapted from Díaz-Reinoso et al. [94].

Subcritical water extraction (SWE) is an emerging approach for the aqueous extraction of lipophilic compounds such as EOs from plant materials (Figure 3). SWE is based on the use of water at a subcritical state (temperatures between 100 and 374.15 °C and pressures below 22.1 MPa) [90]. It is considered a clean extraction method which avoids the consumption of organic solvent and the production of toxic residues. The properties of water show substantial changes in its subcritical state that can be attributed to the reduced intermolecular hydrogen bonds between water molecules. The viscosity of water is decreased and its diffusivity is increased under subcritical conditions. Also, the dielectric constant of subcritical water is comparable to solvents such as methanol, ethanol, acetonitrile acetone, and dimethyl sulfoxide [91,92]. Correspondingly, the polarity of water is reduced under a subcritical state, which increases the solubility of EOs and their compounds [93]. Compared to organic solvents, subcritical water provides increased efficiency, higher extract quality in shorter times, and is a highly available, non-flammable, non-toxic, and easy-to-use solvent [94,95,96]. Therefore, subcritical water can be used as a green substitute to organic solvents for the extraction of EOs such as LEO. In a study by Giray et al. [97], the chemical composition of *Lavandula stoechas* L. EOs extracted by SWE was compared to those obtained by HD and ultrasound-assisted solvent extraction (UASE). SWE was performed by a laboratory-built apparatus at a pressure of 60 bar and temperatures of 100, 125, and 150 °C for 30 min. For HD, the lavender flowers were submitted to extraction for 3 h in a Clevenger-type apparatus. Meanwhile, the UASE was performed in an ultrasound bath with t tert-butylmethyl ether as solvent. The number of individual compounds detected in SWE, HD, and UASE were 124, 94, and 65 as estimated by GC–MS spectrometry. The major components in all of the EOs were camphor, fenchon, myrtenol, myrtenyl acetate, and 1,8-cineol, but they differed in quantity. The EOs extracted by SWE contained higher amounts of light- and heavy-oxygenated compounds compared to the EOs obtained by HD and UASE. However, lower amounts of cyclic monoterpenes were detected in the EOs extracted by subcritical water. The temperature of subcritical water had a significant effect on the extraction yield and the LEO yield was increased with the increase in temperature; however, its ability to extract more polar compounds such as camphor, 1,8-cineol, and fenchon was decreased. The GC–MS analysis results suggested that the optimum extraction temperature for the SWE of LEO is 100 °C. The kinetic studies demonstrated that SWE is obviously quicker than HD and UASE; the SWE was completed in 55 min, while 3 h was required for HD and 18 h for UASE to completer the extraction process. Also, the LEO extracted by SWE was more concentrated and had a more powerful aroma compared to those extracted by HD and UASE. In a similar study, Eikani et al. [98] performed a comparative study on *Lavandula latifolia* Medik EO extraction by SWE, HD, and Soxhlet extraction. A laboratory-built subcritical water extractor was filled with lavender (4.0 g) and the influence of temperature (100–175 °C), pressure (20–40 bar), and flow rate (1–4 mL/min) on the extraction yield and composition of LEO was studied. HD was carried out in a Clevenger-type apparatus with 4.0 g of dried plant material and 150 mL water for 3 h. While, Soxhlet extraction was performed in a standard Soxhlet apparatus containing 4.0 g lavender and 200 mL hexane, and the extraction process took 12 h. The separation and identification of extracted compounds were carried out using GC-FID and GC/MS. The optimum operating conditions in the SWE process included 150 °C, 20 bar and 3 mL/min. Under these conditions, the extraction yield was similar to that of HD and Soxhlet extraction. However, SWE was carried out in a shorter time (30 min) and was more selective for the extraction of oxygenated compounds. Based on these studies, SWE has a great potential to be used as a green extraction approach for LEO. However, the processing temperature and time should be selected carefully to avoid the degradation of valuable compounds. Also, caution is required for safe operation at high pressure and high temperature, and the environmental effects must be considered [94].

#### 3.2.3. Microwave-Assisted Extraction

**Figure 4:**
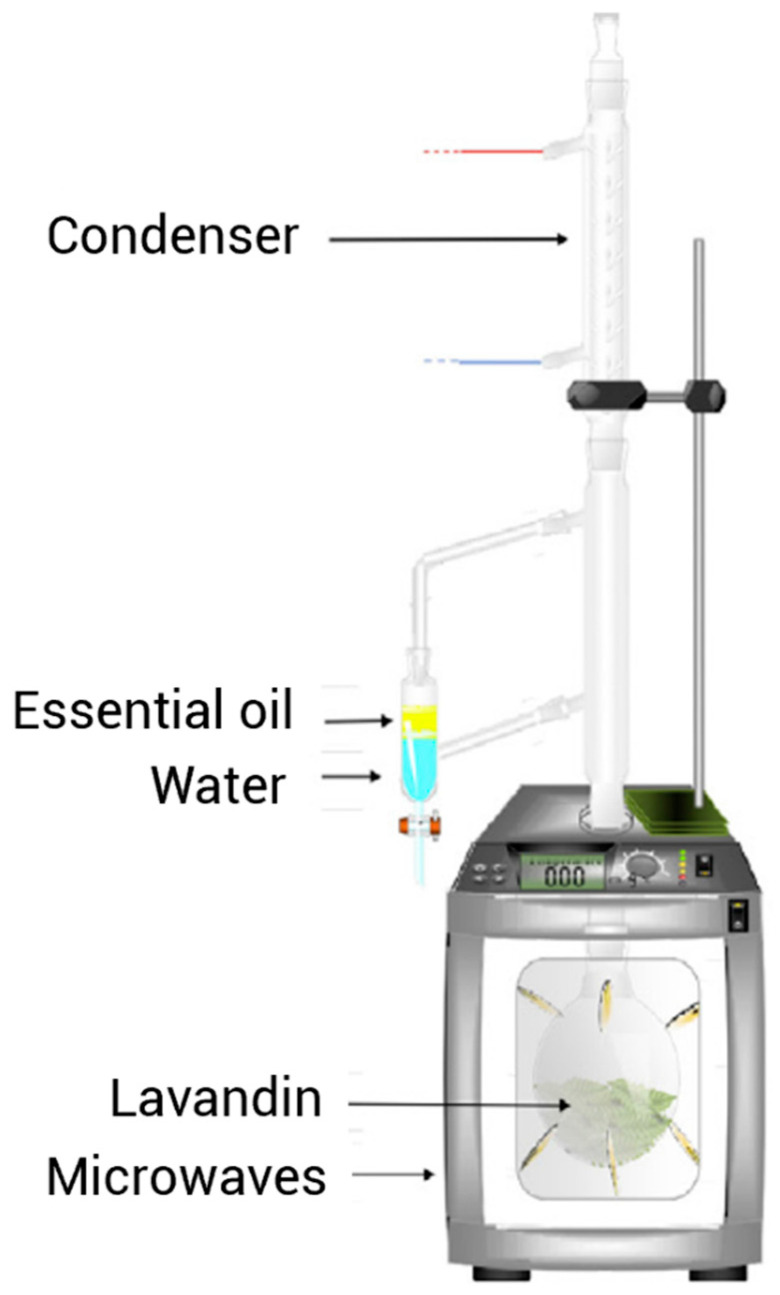
A schematic diagram of the microwave-assisted steam distillation (MASD) system; adapted from Périno-Issartier et al. [108].

Microwaves are electromagnetic waves with frequencies ranging from 300 MHz to 300 GHz. Microwave energy is a non-contact heat source for the extraction of different compounds from plant materials. Microwave energy can significantly increase the extraction rate of different compounds such as EOs, which typically takes hours to complete the extraction process in conventional methods. MAE (Figure 4) can be completed within minutes with high reproducibility, reduced waste water, solvent consumption, thermal gradients, and equipment size, higher final product purity, faster energy transfer, and consuming a much smaller amount of energy compared to the conventional method [99,100]. MAE is based on two phenomena, namely dipole rotation and ionic conduction, that usually occur simultaneously [101]. The former is the realignment of ions under an electromagnetic field that increases the temperature of extraction medium, while the latter is caused by the electrophoretic migration [102,103].

Microwaves cause the quick rupture of glandular walls and increase the quality and quantity of EOs. Advances in this extraction method have led to the development of techniques such as microwave-assisted/accelerated hydro-distillation (MAHD), microwave-assisted/accelerated steam distillation (MASD), microwave steam diffusion (MSD), solvent-free microwave extraction (SFME), microwave-assisted solvent extraction (MASE), vacuum microwave hydro-distillation (VMHD), compressed air microwave distillation (CAMD), and microwave hydrodiffusion and gravity (MHG) [99]. Several studies have revealed that MAE is an efficient approach for LEO extraction owing to its high yield and short extraction time, as well as its low solvent rate and cost (Table 2). Filly et al. [93] extracted LEO by ten extraction techniques including SD, HD, salt–hydro-distillation (NaCl-HD), turbo-hydro-distillation (THD), micelle–hydro-distillation (Micelle-HD), enzyme–hydro-distillation (Enzyme-HD), ultrasound–hydro-distillation (US-HD), subcritical water–hydro-distillation (SW-HD), and SFME. The experimental variables such as the amount of salt, surfactant, enzyme, and microwave power for each extraction method were optimized to obtain the maximum LEO yield. The extraction yield of LEOs and their chemical compositions were compared with each other. Also, the extraction time, CO_2_ emission, and energy consumption were determined and compared with HD. The obtained results suggested that MSD is the most appropriate method for the extraction of LEO, as it reduced the formation of by-products and resulted in higher oil recovery in a shorter time. The yield of HD was 4.6% in 120 min, while MSD produced an extraction yield of 5.4% in 30 min. Additionally, the energy consumption of MSD was 0.298 kWh, while it was 3.452 kWh in conventional HD.

Danila et al. [104] compared the extraction time, yield, and chemical composition, of LEO extracted by HD and MAE. The time of extraction was shorter in MAE compared to HD and the yield was higher, as the extraction yield was 0.124% for HD and 0.6% for MAE. The GC-MS analysis identified 44 components in the LEO extracted by HD and 46 components in the EOs extracted by MAE. The major compound of both LEO samples was linalool; however, the other main compounds were completely different. González-Rivera et al. [105] used a microwave-assisted Clevenger-type apparatus and a conventional hydro-distillator to extract EOs from different plants including lavender. The extraction yield and composition of EOs extracted by conventional HD and coaxial microwave were studied as a function of extraction time. Water and plant material were mixed in a ratio of 5:1 and the dispersion was placed into a flask vessel in which the microwave energy was applied to the extraction medium with a coaxial antenna. The dispersion was subjected to a microwave irradiation power of 500 W and heated for 9 min while stirring at 250 rpm. As the distillation started, the microwave power was reduced to 300 W and kept constant for different times (5–60 min) to investigate the kinetics of extraction process. In conventional HD, the prepared dispersions were heated using a standard electric mantle operating at 250 W. The extraction time was 180 min in conventional HD, while the microwave extraction with the coaxial antenna reduced the extraction time to 30 min. Also, higher concentrations of valuable oxygenated monoterpenes were extracted in this process compared to conventional HD. For instance, the amount of 1,8-cineole in the LEO extracted by coaxial microwave was 44.4%, while it was 33.0% for conventional extraction. The microwave-accelerated SD (MASD) and conventional SD of LEO were compared by Chemat et al. [106]. In the MASD process, steam was produced by a microwave reactor operating at 2.45 GHz and 500 W. The apparatus consisted of a cylindrical Pyrex body with a Teflon grid at its lower end. Steam was generated in the lower part of the apparatus and passed through a bed of dried lavender flowers to extract the EOs. Then, it was directed to the condenser on the top of the instrument to separate LEO and water. In the SD, a similar glassware was used, but steam was generated by an electrical resistance heater. The results revealed that MASD resulted in higher LEO yield (8.86% vs. 8.75%) and oxygenated monoterpenes (78.29% vs. 75.14%), and reduced extraction time (10 min vs. 90 min) compared to SD. These findings are in agreement with the results reported by Calinescu et al. [107], who reported that the LEO extracted by microwave-assisted HD contains higher amounts of oxygenated compounds. They also found that an enzymatic pretreatment of lavender flowers can significantly reduce the extraction time while increasing the LEO yield and quality. However, some studies demonstrated that the extraction yield and composition of EOs produced by MAE are comparable to those obtained by conventional extraction approaches. Périno-Issartier et al. [108] used different conventional and emerging extraction methods to extract EOs from the inflorescence of lavandin and compared the quality and quantity of the extracted EOs. The conventional methods included HD, SD, and turbo-HD, while emerging techniques included ultrasound-assisted extraction (UAE) as a pretreatment and extraction with different microwave-assisted extraction approaches, such as MSD, in situ microwave-generated hydro-distillation (ISMH), and MHG. The EOs extracted by innovative and conventional methods presented similar yields and chemical compositions. However, the maximum extraction yield (5.4%) was observed after 15 min of MHG, while it took 120 min in SD. Also, the energy consumption was 1.3 kWh in MHG against 8.06 kWh in SD. Therefore, MHG was identified as the best extraction technique with the shortest extraction time and the lowest energy consumption. In another study, Farhat et al. [99] used MSD for LEO extraction and compared the results with SD. The EOs obtained by the MSD method were qualitatively and quantitatively similar to those extracted by SD. The efficiency of MSD was confirmed by a mathematical model and it was found that the solid–steam mass transfer coefficient in MSD was six times greater than that of SD. The MSD method reduced the amount of waste water and energy consumption; thus, it is more environmentally friendly compared to SD. Likewise, Sahraoui et al. [109] reported that the MASD and conventional SD of lavender flowers resulted in similar LEO yields and aromatic profiles. However, the MASD is preferred to SD due to its rapidity (6 min vs. 30 min). In another study, Iriti et al. [110] compared the EO yield and the structure of glandular trichome of *Lavandula angustifolia* flowers extracted by conventional HD, MAHD, and MASD. Conventional LEO extraction was carried out by a Clevenger for 4 h with 80 g of dried lavender flowers and 4 L of water. In MAHD, the flowers (80 g) were soaked in distilled water (1.5 L) for 15 min and then the water was removed and extraction was performed by a microwave oven at atmospheric pressure for 20 min at 500 W. The MASD process was the same as MAHD, but 200 mL of water was kept under a perforated Teflon plate, on which lavender flowers were loaded. The quantity and chemical composition of LEO extracted in 20 min of MAHD and MASD were relatively similar to that obtained by HD after 4 h. The SEM images of glandular trichome after 4 h of HD exhibited a deeply wrinkled surface that preserved the integrity of the cuticle but was devoid of EOs. After 20 min of microwave-assisted extraction, the glandular trichomes appeared empty as well, but they were predominantly disrupted, or at least there were holes in their head cuticle. These findings suggest that the high rate of microwave-assisted extraction in EO extraction is primarily due to the destruction of the trichome structure that facilitates the release of its content, while in conventional HD the EOs slowly penetrate through membranes and cuticles. Liu et al. [111] optimized the MAHD extraction parameters by RSM coupled with Box–Behnken design for LEO extraction and compared the results with HD. The effects of extraction parameters such as liquid/solid ratio (6–22 mL/g), microwave power (400–700 W), and extraction time (10–40 min) on LEO yield were examined in single-factor experiments. The optimized conditions of MAHD were as follows: a liquid/solid ratio of 17 mL/g, a microwave power of 500 W, and a microwave time of 40 min. The results indicated that microwave time had the highest effect on the LEO yield, followed by liquid/solid ratio and microwave power. The LEO yield was 3.19% after 40 min of extraction under the optimized conditions of MAHD, which is approximately similar to the predicted value (3.20%) and the extraction yield in HD (3.23) after 120 min. Therefore, MAHD is superior in terms of extraction time and energy saving. The GC-MS analysis of LEO samples obtained by HD and MAHD did not show obvious differences, and both EOs presented 39 compounds. However, the antimicrobial tests revealed that the LEO extracted by MAHD has a greater antimicrobial activity compared to the hydro-distilled oil. Based on the available data and published papers, MAE offers higher extraction yields in shorter times and has great potential to be the main innovative approach for the industrial extraction of EOs in the near future. However, the industrial scale-up of microwave extractors is not an easy task, due to the low irradiation depth of microwaves, the irregular distribution of electromagnetic field, the non-uniformity of irradiation, and the formation of hot-spots. These problems hinder the industrial application of microwave energy for the extraction of EOs and should be addressed in future studies.

**Table 2:** The effects of species, cultivation area, and microwave-assisted extraction conditions on the yield and composition of lavender essential oil.

Species	Cultivation Area	Extraction Conditions				LEO Yield (%)	Main Components	Reference
		Power (W)	T (°C)	Time (min)	Solid/Liquid Ratio			
*Lavandula angustifolia*	Bergamo, Italy	500	100	10	1:4	8.86	Linalool and camphor	[106]
*Lavandula angustifolia*	Prahova, Romania	2000	96	15	1:2	0.6	Linalool and β-Fenchyl acetate	[104]
*Lavandula angustifolia*	Avignon, France	200	100	3	NS	4.42	Linalool and 1,6-Octadien-3-ol, 3,7-dimethyl, acetate	[99]
*Lavandula angustifolia*	Tuscany, Italy	500	NS	30 min	1:5	0.75	1,8-cineole and camphor	[105]
*Lavandula angustifolia*	Bergamo, Italy	500	100	20 min	1:2.5	7.70	Linalool and camphor	[110]
*Lavandula angustifolia*	Province, France	200	NS	6 min	NS	2.7	Linalool and 1,8-Cineole	[109]
*Lavandula coronopifolia* Poir.	Niakan Village, Iran	800	NS	60	1:20	0.63	Dodecamethylpentasiloxane and tetracosamethylcyclododecasiloxane	[112]
*Lavandula x intermedia* var. Super A	Isparta, Turkey	750	NS	120 min	1:10	5.5	Linalool and 1,8-cineole	[29]
NS	Nottingham, United Kingdom	175	100	11.57	1:2	1.87	NS	[113]
NS	Xinjiang, China	500	NS	40 min	1:17	3.16	Linalool and linalyl acetate	[111]
NS	Beijing, China	NS	105	120	1:12	3.67	Formic linalool ester and terpineol	[70]

T: temperature; NS: not stated.

#### 3.2.4. Ultrasound-Assisted Extraction

Sonication is an acoustic treatment by means of high-frequency sound waves. The frequency of ultrasound waves is higher than 20 kHz, which is beyond the threshold of human hearing. In UAE, the plant materials are mixed with water or other solvents and exposed to sonication [114]. The travel of ultrasound waves within the solvent produces alternating compressions and expansions that form pressure change regions and cause the formation of cavitation bubbles in the aqueous phase. The cavitation phenomenon facilitates the flow of the liquid phase inside the plant cells, and thus, increases the efficiency of EO extraction [115]. Also, the collapse of cavitation bubbles increases the temperature and pressure up to 4000 K and 1000 atm, respectively, which induce cell damage and facilitate the release of EOs [116]. Lilia et al. [117] evaluated the effect of ultrasound pretreatment on the extraction yield, antioxidant activity, and composition of LEO extracted by HD with a Clevenger-type apparatus. The EOs were extracted from Adekar and Keddara, two wild varieties of *Lavandula stoechas* L. The results showed a rapid release of EOs from both lavender varieties. The EO recovery of Adekar variety was 1.59% after 10 min of sonication pretreatment and 90 min of HD, while the EO recovery for the untreated sample was 1.17% after 180 min of HD. On the other hand, the EO recovery from Keddara variety was 0.62% in the untreated sample and increased to 0.87% after ultrasound treatment. Also, the chemical composition of the EOs was affected by the lavender variety and the extraction method, and the concentration of LEO individual compounds was increased by sonication. Consequently, the antioxidant power of sonicated samples was enhanced. Coupling ultrasound and other emerging technologies such as the microwave increases the extraction yield through the disintegration of plant tissue and facilitates the flow of liquid phase into the glandular trichomes [115]. Sharifzadeh et al. [112] used ultrasound as a pretreatment to increase the microwave-assisted extraction yield of LEO. The process was called the sequential ultrasonic microwave-assisted extraction (SUMAE) method. The extraction yield, chemical composition, antioxidant properties, antimicrobial activity, and environmental effects of the SUMAE process were determined and compared with MAE method. Parameters such as microwave power, ultrasound power, and extraction time were the independent variables for the optimization of extraction conditions. The LEO yield was considered as the response and the optimization was performed by RSM using a central composite design (CCD). For the SUMAE, the plant material and distilled water were blended in a ratio of 1 to 10 and sonicated for 20 min. Subsequently, the mixer was heated for 20 to 40 min and 600 to 800 W in a microwave heater connected to a Clevenger, while in the MAE, the EOs were extracted without sonication pretreatment. The highest LEO yield (1.15% *w*/*w*) was obtained by 800 W of microwave power and 150 W of ultrasound power after 40 min of extraction. The LEO yield was increased by approximately 82% in SUMAE compared to the MAE method. According to the GC–MS analysis results, the number of LEO components was increased in the SUMAE compared to MAE. Moreover, the DPPH and ferric-reducing antioxidant power (FRAP) essay results showed higher antioxidant properties of the EOs extracted by SUMAE. The antimicrobial tests indicated that the EOs obtained from SUMAE have higher antimicrobial activity. Rashed et al. [115] employed RSM to optimize the processing conditions for LEO extraction by ultrasonic microwave-assisted approach and enzymatic pretreatment with cellulase and hemicellulose. Dried and ground lavender inflorescence (100 g) was immersed in distilled water (500 mL) containing varying amounts of single or combined enzymes. The samples were placed in a shaking water bath at 40 °C for 1 h and then the extraction of LEO was implemented in an ultrasonic microwave cooperative extractor connected to a Clevenger-type apparatus for 3 h. The control LEO sample was extracted under the same conditions without enzymatic pretreatment. The process variables for the extraction optimization included the enzyme concentration (5–20 mg), microwave power (500–50 W), and sonication time (15–90 min). The highest yield of LEO was obtained by a sonication time of 52.5 min, a microwave power of 275 W, and an enzyme concentration of 12.5 mg. Also, it was found that the LEO yield was increased by combined enzymes (cellulase and hemicellulase). Generally, the published papers indicate that the UAE technology holds promise for the extraction of plant materials such as EOs while maintaining their quality.

#### 3.2.5. Pulsed Electric Field Extraction

Pulsed electric field (PEF) is an innovative technique based on using intense electric pulses for a very short period of time (milliseconds to microseconds). PEF equipment is composed of a treatment chamber, a high-voltage DC power supply, an energy storage capacitor, and a spark gap switch. The high-voltage source charges the capacitors until the spark gap breaks down, thereby causing a sudden voltage (shock) to the treatment chamber into which the sample is loaded [118,119]. PEF can be used as a pretreatment to improve the extraction of bioactive compounds from plant materials. It has been stated that PEF electroporates the biological membranes and disrupts the plant cells. Consequently, the release of EOs is facilitated and the extraction rate is improved [120]. In this regard, Hadri et al. [121] studied the effect of PEF pretreatment on LEO extraction. Water and plant material were mixed in a ratio of 3:1 and placed in the treatment chamber of PEF instrument which were composed of two stainless steel electrodes with a gap of 1.5 cm, and the extraction was accomplished by HD with a Clevenger-type apparatus. The effects of voltage, pulse number, and distillation time on LEO extraction yield and composition were investigated. The results revealed that PEF treatment significantly improved the extraction of LEO. The LEO yield of the PEF-pretreated sample was 3.0% after 30 min of HD and 4.0% after 60 min of HD, while it was only 2.95% after 60 min of HD without PEF treatment. PEF treatment at 1 kV/cm with 100 pulses for 60 min constituted the optimum extraction conditions. Also, the energy consumption was reduced approximately 50% after PEF pretreatment. The DPPH analysis results indicated that the EOs have high antioxidant activity. Also, GC-MS analysis revealed that the EOs obtained from PEF-pretreated lavender were of good quality and contained the components that can be decisive in medicinal properties and industrial application. Therefore, PEF can be applied to aromatic plants in order to produce higher amounts of EOs within a significantly shorter distillation time.

#### 3.2.6. Cold Plasma Extraction

**Figure 5:**
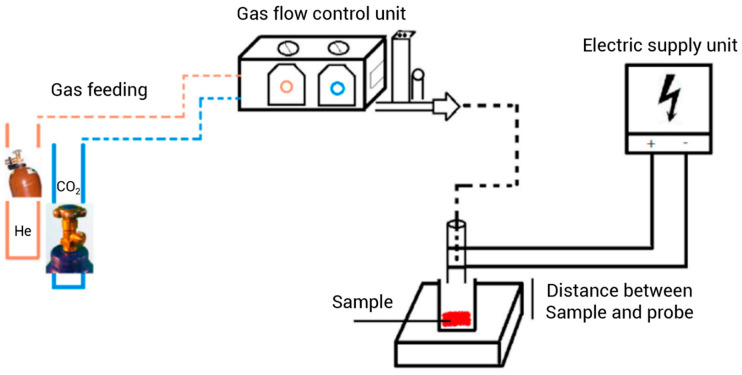
A schematic diagram of the dielectric barrier discharge (DBD) plasma system; adapted from Ucar et al. [127].

Plasma is the fourth state of matter that exists naturally in the universe (e.g., the polar aurora) but can be produced artificially by electrical discharges in a gas. Plasma is composed of reactive oxygen species, reactive nitrogen species, ultraviolet (UV) radiation, free radicals, and charged particles. It is an ionized gas that can be classified into thermal plasma (TP) and cold plasma (CP). In TP, all species exist in a thermodynamic equilibrium, while in the CP, the electrons and heavier species are in thermal non-equilibrium [122]. Based on the pressure conditions, plasma can be categorized into atmospheric pressure, high-pressure, and low-pressure plasma. Plasma can be generated from gases such as oxygen, ozone, nitrogen using different sources of energy including thermal, electrical, optical, electromagnetic, and gamma radiation [123]. Plasma jet and dielectric barrier discharge (DBD) are the most widely used plasma-generating devices (Figure 5). The plasma jet system comprises two concentric electrodes, where the inner electrode is connected to a radio frequency power, ionizing the working gas that exits the nozzle, giving it a “jet-like” appearance. In contrast, DBD consists of two metal electrodes, at least one of which is covered with a dielectric barrier. The dielectric barrier acts as a stabilizing material that prevents any arc transfer, and helps create a large number of micro-discharges for homogeneous treatments. When a material is exposed to plasma treatments, the reactive species can modify its surface by the formation of hydrophilic groups or oxidation. These modifications are limited to the surface of the plasma-treated material and do not affect their internal properties. Also, small cracks and fissures may appear in the epidermal cell structure. The formation cracks and hydrophilic groups improve the EO extraction from aromatic plants [124,125]. Therefore, CP can be used to increase the extraction of phytochemicals such as EOs. The application of CP treatment for EO extraction is a novel approach and its effects on EO composition and properties are not fully understood. Consequently, in-depth studies are required to determine the optimum processing conditions and the possible interactions between the plant materials and CP. Molina et al. [126] explored the effect of CP treatment on the extraction yield of lavandin EO. CP was generated by a dielectric barrier discharge reactor consisting of two aluminum electrodes with glass covers that create a 1 cm gap. The lavandin flowers were located on the lower electrode of the reactor to cover almost the entire electrode area and plasma discharge was initiated. CP treatment was performed at 40W for 1 to 10 min and then the EOs were extracted by HD. Lavandin flowers (100 g) and distilled water (1200 mL) were added to a round-bottom flask and heated in a heating mantle for different times (5 to 120 min). The GC/MS and ATR-FTIR analyses indicated that CP treatment did not significantly affect the composition of LEO and preserved their aromatic compounds, whereas the surface chemistry of lavandin flowers was altered by CP treatment. The surface carbon was decreased and oxygen content was increased, leading to an increase in hydrophilic groups. These hydrophilic groups increased the interactions between the surface membrane of glandular trichomes on lavandin flowers and water vapor; as a result, the LEO extraction was increased. Moreover, the SEM images revealed that CP treatments did not have obvious effects on the glandular trichomes, indicating that the surface changes occurred at the nano scale. The LEO yield was 3.19% in the control sample and increased to 3.44 after 1 min of CP treatment, while the use of CP for 10 min showed the diminishing return of extraction yield to 3.07%. The results underlined the significance of CP treatment as a low-cost and environmentally friendly technology in improving the extraction yield of EOs, reducing the solvent, extraction time, and energy consumption compared to the conventional extraction methods. However, the composition of CP-treated EOs is affected by the treatment time, intensity, and the type of gas used in CP processing. Therefore, future studies should focus on the effect of CP on the phytochemicals and optimization of CP processing conditions to produce EOs with great aromatic profiles and prevent the generation of hazardous compounds.

#### 3.2.7. Ohmic-Assisted Extraction

**Figure 6:**
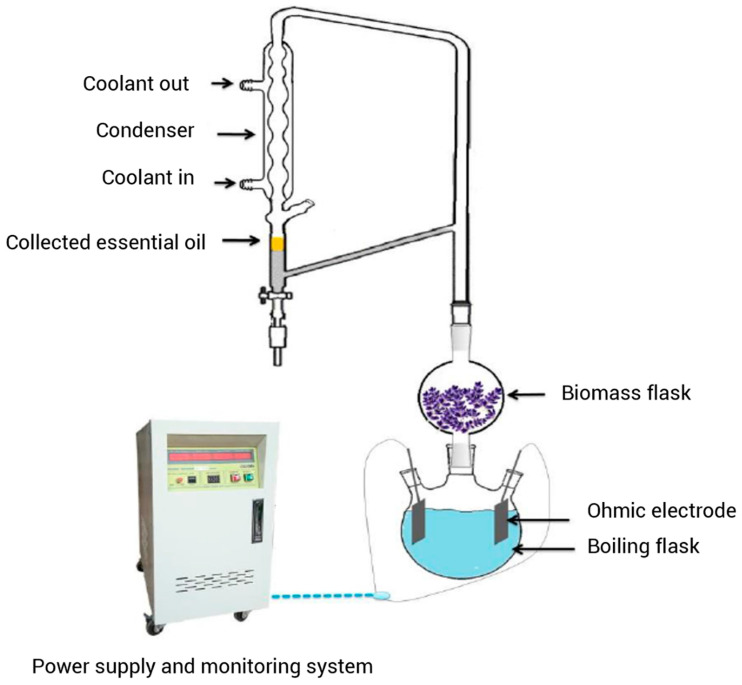
A schematic diagram of the ohmic-accelerated steam distillation (OASD) system; adapted from Gavahian and Chu [132].

Ohmic heating (OH) is an emerging thermal process that generates heat within the food matrix [128]. The OH is referred to as a high-temperature short-time (HTST) process, during which an alternating current (AC) is passed through the food and increases its temperature uniformly due to electrical resistance. Accordingly, the disadvantages of classical heating systems such as the contamination of heat exchangers, overheating, and non-uniform heating of food do not occur in OH. This emerging technology is used in various fields of food processing such as extraction, sterilization, blanching, evaporation, dehydration, and fermentation owing to its high efficiency in inactivating microorganisms, shorter processing time, and less energy consumption compared to conventional methods [129,130]. Furthermore, it has also been found that food products processed with OH have higher organoleptic and nutritional quality. In recent years, the use of OH to extract EOs has emerged as a promising approach, and several researchers have successfully used OH to extract EOs from aromatic plants. However, most of these studies have extracted EOs by an ohmic-assisted hydro-distillation (OAHD) process in which the plant material is in contact with the electrodes that may cause electrochemical reactions and have negative effects on the phytochemicals. In an ohmic-assisted/accelerated steam distillation (OASD) system, steam is generated by an ohmic heater and then passes through a bed of plant material (Figure 6). Therefore, the plant material is separated from ohmic electrodes. This system can eliminate the possible adverse effects of electrochemical interactions and electrode corrosion on bioactive compounds and enable the exploration of a range of electrical frequencies, low-cost electrode materials, and higher electrolyte concentrations [131].

In this regard, Gavahian and Chu [132] designed an OASD system to extract LEO and compared the properties of the extracted EOs with those extracted by SD. The OASD apparatus consisted of a boiling flask that was equipped with two rectangular (4 × 3 cm) titanium electrodes. The flask was filled with NaCl solution of 0.5%, because salted water has a higher electrical conductivity compared to pure water and is more efficient in generating steam. The steam produced in the boiling flask passed through the flask that contained 30 g of lavender flowers. The volatile compounds were extracted and placed in the Clevenger apparatus section to cool and condense the evaporated LEO. Conventional SD was performed with the same amounts of lavender and salted water; however, an electric mantle heater was placed at the bottom of the boiling flask to generate steam. The results revealed that the extraction yields of both techniques are similar with the same IC50 values (100 μg.mL^−1^). Also, the GC-MS analysis exhibited that OASD did not have negative effects on the LEO composition and the main compounds of both EOs were linalool, camphor, and 1,8-cineole. In addition, the color parameter values and the relative densities of extracted EOs were similar. OASD was much faster than SD; the extraction time was 50.3 ± 2.1 min in OASD and 112.7 ± 5.9 min in SD. Also, the energy consumption of OASD was 0. 4 KWh, while it was 0.9 KWh in SD for the extraction of 1 mL LEO. Future studies should focus on process optimization, economic analysis, and large-scale studies to evaluate the applicability of OASD and pave the way for its industrial adaptation.

## 4. Research Gaps and Future Trends

At present, research on the extraction of LEO has made notable advancements; nevertheless, several research gaps remain that offer opportunities for further exploration and innovation. Firstly, although conventional extraction methods, e.g., SD, are prevalent, there is an immediate necessity to simplify and modify these processes in order to improve their efficiency and sustainability. In addition, delving into variations in extraction parameters, such as pressure, temperature, and duration, can provide valuable insights into optimizing oil yield and quality. Secondly, the utilization of emerging technologies for the extraction of LEO is a developing field with considerable potential. In this respect, various methods, such as supercritical fluid extraction (SFE), organic solvent-assisted extraction (OAE), MAE, and UAE, among others, have demonstrated several advantages, yet their scalability, cost efficiency, and relative efficacy compared to traditional methods require further comprehensive investigations. In addition, understanding the underlying mechanisms of these emerging technologies and their possible impacts on the chemical composition of the extracted EOs is essential for their successful industrial applications. On the other hand, while there is growing interest in sustainable methods for the extraction of LEO, there remains a significant gap in comparing the environmental impact and economic feasibility of conventional and emerging extraction techniques. In this regard, it is crucial to evaluate the possible ecological footprint of different extraction processes, as well as their cost effectiveness, scalability, and market competitiveness to incentivize their adoption by industry stakeholders. Moreover, different lavender species and varieties may have a significant effect on the yield and chemical profile of the extracted EOs, thereby affecting their suitability across diverse industries, such as food, pharmaceuticals, and cosmetics. Hence, a thorough examination of these variations can help the selection of lavender cultivars tailored to specific end-use requirements. Furthermore, investigating various post-extraction processing aspects, such as the stability, shelf life, and potential degradation pathways of LEOs under different storage conditions is vital for preserving their quality and bioactivity over time. In summary, addressing these research gaps in future studies can contribute to the progress in LEO extraction technologies, enhancing yield and efficiency, strengthening sustainability, and expanding the scope of their applications across various industries.

## 5. Conclusions

LEO is a highly consumed bioactive compound with great economic value. It can be used in foods, textiles, drugs, pharmaceuticals, cosmetics, and agro-biological products as a natural flavor, antioxidant, preservative, and therapeutic agent. The extraction methods and conditions have substantial effects on the physicochemical and biological properties of LEO. SD and HD have been frequently used for LEO extraction. However, these techniques are not time- and energy efficient and may damage the bioactive and aromatic compounds of LEO. The limitations of conventional extraction methods have led to the development of novel extraction technologies. The extraction of EOs by emerging technologies such as ultrasound-assisted, microwave-assisted, ohmic-assisted, cold plasma, pulsed electric field, and sub- and super-critical fluid extraction methods has gained popularity in recent years. Emerging technologies promote EO recovery, minimize solvent use, and reduce time and energy consumption, while preserving the chemical integrity of LEO. Therefore, they have a lower environmental impact compared with conventional methods and are considered green and sustainable extraction methods. Overall, this overview of published papers underlines the promising advancements of emerging technologies that would help to save cost, time, and effort of LEO extraction. However, scale-up studies and the optimization of industrial operating conditions should be performed in order to determine the industrial scalability of LEO extraction by emerging technologies.

## Data Availability

No new data were created or analyzed in this study. Data sharing is not applicable to this article.
